# Mild Hypoxia Enhances Proliferation and Multipotency of Human Neural Stem Cells

**DOI:** 10.1371/journal.pone.0008575

**Published:** 2010-01-05

**Authors:** Guido Santilli, Giuseppe Lamorte, Luigi Carlessi, Daniela Ferrari, Laura Rota Nodari, Elena Binda, Domenico Delia, Angelo L. Vescovi, Lidia De Filippis

**Affiliations:** 1 Department of Biotechnology and Biosciences, University of Milano-Bicocca, Milan, Italy; 2 Fondazione IRCCS Istituto Nazionale Tumori, Department of Experimental Oncology, Milan, Italy; City of Hope National Medical Center, United States of America

## Abstract

**Background:**

Neural stem cells (NSCs) represent an optimal tool for studies and therapy of neurodegenerative diseases. We recently established a v-myc immortalized human NSC (IhNSC) line, which retains stem properties comparable to parental cells. Oxygen concentration is one of the most crucial environmental conditions for cell proliferation and differentiation both in vitro and in vivo. In the central nervous system, physiological concentrations of oxygen range from 0.55 to 8% oxygen. In particular, in the in the subventricular zone niche area, it's estimated to be 2.5 to 3%.

**Methodology/Principal Findings:**

We investigated in vitro the effects of 1, 2.5, 5, and 20% oxygen concentrations on IhNSCs both during proliferation and differentiation. The highest proliferation rate, evaluated through neurosphere formation assay, was obtained at 2.5 and 5% oxygen, while 1% oxygen was most noxious for cell survival. The differentiation assays showed that the percentages of β-tubIII+ or MAP2+ neuronal cells and of GalC+ oligodendrocytes were significantly higher at 2.5% compared with 1, 5, or 20% oxygen at 17 days in vitro. Mild hypoxia (2.5 to 5% oxygen) promoted differentiation into neuro-oligodendroglial progenitors as revealed by the higher percentage of MAP2+/Ki67+ and GalC+/Ki67+ residual proliferating progenitors, and enhanced the yield of GABAergic and slightly of glutamatergic neurons compared to 1% and 20% oxygen where a significant percentage of GFAP+/nestin+ cells were still present at 17 days of differentiation.

**Conclusions/Significance:**

These findings raise the possibility that reduced oxygen levels occurring in neuronal disorders like cerebral ischemia transiently lead to NSC remaining in a state of quiescence. Conversely, mild hypoxia favors NSC proliferation and neuronal and oligodendroglial differentiation, thus providing an important advance and a useful tool for NSC-mediated therapy of ischemic stroke and neurodegenerative diseases like Parkinson's disease, multiple sclerosis, and Alzheimer's disease.

## Introduction

Cultured CNS stem cells are endowed with capacity to self-renew and differentiate into neurons, astrocytes and oligodendrocytes in predictable proportions [1]–[5]. Thus they have provided a useful tool to elucidate the pathways leading to generation of neurons and glia and to study the effects of different extrinsic factors on the commitment of neural stem cells (NSC) to form such cell lineages [6]. For these reasons the discovery, isolation and characterization of multipotent NSC from various locations within the mammalian brain represents a major recent advancement in neuroscience [7].

Inevitably, NSCs have become a hot topic of investigation in translational research for common degenerative diseases. In fact, an important goal is to accomplish neuroregeneration by transplantation of exogenous cells that is by cell-mediated therapy. In clinical settings, gases are appreciated as primary variables in organ survival, with O_2_ as the critical gas parameter. Indeed, oxygen plays an essential role in the maintenance of NSC viability as it is responsible for aerobic metabolism to maintain intracellular energy balance. Hypoxia, as a state of reduced O_2_ tension below critical values, triggers intricate and complex mechanisms to restore O_2_ homeostasis at the cellular, tissue and organism level and it occurs under physiological as well as pathological conditions. Markedly severe hypoxia (less than 0.002% O_2_) is caused pathologically by stroke, ischemia and increase in solid tumor size [8], [9].

Cerebral ischemia is known to cause acute and delayed neuronal death through the activation of a complex series of events leading to severe brain dysfunction both in rodents and humans [10], [11]. Recent studies have shown that both global and focal ischemia induce increased proliferation and neural differentiation of NSCs residing in the subgranular zone (SGZ) of the dentate gyrus (DG), the anterior subventricular zone (SVZ) and the posterior periventricular zone adjacent to the hippocampus [12], [13]. A parallel increase of the migration of NSCs along the neurogenic pathways was also observed [14], but the mechanisms involved are still unknown. Hypoxia is among the main factors causing ischemia-derived injuries. The physiological concentration of oxygen in the central nervous system (CNS) ranges from as low as 0.55% in the midbrain to 8% in the pia [15]. In particular, 3–5% oxygen enhances the proliferation of cultured NSCs and modulates their differentiation into neurons [16], [17].

We have established an immortal human NSC line (IhNSC) cultured at 5% oxygen that retains normal hNSC features such as proliferation, self-renewal and multipotency [18]. In particular, IhNSC can generate fully functional neuronal cells, thus providing a useful model to study NSC for the therapy of neurodegenerative diseases or brain injuries like stroke and ischemia, without the limitations of primary fetal tissue. We recently transplantated IhNSC-derived progenitors (IhNSC-P) near the hippocampal CA1 layer of adult rats injured by global transient ischemia to evaluate the integration and maturation of hNSC in a context mimicking the chronic impairment of neurological function following hypoxia-induced injuries, and documented their ability to engraft efficiently, to the point of establishing synaptic contacts with the host cells (Rota Nodari et al, submitted).

Considering these findings, we have examined the effects of different oxygen concentrations (1%, 2.5%, 5% and 20% atmospheric oxygen) on the proliferation, differentiation and death of IhNSC in order to identify the optimal culture conditions of non-immortalized human NSCs for NSC-mediated therapy of CNS injuries characterized by severe hypoxia-associated cell death that occurs in stroke and ischemia.

## Materials and Methods

### Generation and Expansion of the IhNSC Line

PK-VM-2 is a replication-defective, infective retroviral vector described previously [1], [19]–[21] coding for both avian *myc* (p110 gag-myc or v-*myc*) driven by the MoLV-long terminal repeat (LTR) promoter and aminoglycoside transferase (conferring resistance to neomycin; neor) driven by the SV40 promoter. The amphotropic retroviral particles were packaged in GP envAM cells cultured in hNSC medium [20]. The hNSC cultures (parental cells) used in this study were isolated and propagated from the diencephalic and telencephalic brain regions of a caucasian human fetus at 10.5 weeks gestational age and were previously described by Vescovi et al. [1]. Retroviral transduction with v-*myc* was carried as described in Villa et al. [21] on parental hNSCs that had undergone 22 passages *in vitro*. Following G418 selection, aliquots of these bulk cell lines were cryopreserved in complete medium containing 10% dimethyl sulfoxide.

### Generation of Growth Curves

hNSC lines 1 and 2 were established by fetal human brain as described [1]; at passage 14 and 16 respectively, they were shifted to appropriate oxygen culture conditions. IhNSC were cultured as hNSC in 5% oxygen and at passage 45 they were split to 1%, 2.5%, 5% and 20% oxygen. In order to allow the adaptation to the new oxygen concentrations, hNSC and IhNSC were cultured for 4–5 passages after the shift before use for the experiments. Thereafter, hNSC and IhNSC were continuously maintained in the respective oxygen concentrations. hNSC and IhNSC were cultured in four different humidified CO_2_ multigas incubators (Binder), flushed continuously with a N_2_ gas to maintain established atmospheric O_2_ concentrations (1%, 2.5%, 5% and 20%) at a constant temperature of 37°C.

The rate of expansion of IhNSC and hNSC was obtained by plating 1×10^4^ cells/cm^2^ in growth medium containing FGF2 and EGF (Peprotech, Rocky Hill, NJ) and grown in the described oxygen conditions. At each passage (p), NSC-originated neurospheres were dissociated and the logarithmic value of the total viable-cell number was plotted against the day *in vitro* (days) since the beginning of the experiment. For each oxygen condition, the growth curves were performed in duplicate and generated comparable results.

### Differentiation of IhNSC

To induce IhNSC differentiation, individual spheres were mechanically dissociated and transferred onto laminin (Roche, Basel, Switzerland) coated glass coverslips at a density of 1×10^4^ cells per cm^2^ in the presence of FGF2 (20 ng/mL). At 3 days, FGF2 medium was replaced with control medium and IhNSC differentiated for additional 7 days (10 days) or 14 days (17 days).

### Immunofluorescence

Differentiating cells were fixed in freshly buffered 4% paraformaldehyde at 3, 10 or 17 days along differentiation (see “Differentiation of IhNSC”). After blocking with 10% normal goat serum and treatment with Triton X-100, 0,3% v/v for intracellular antigen detection, cultures were incubated overnight at 4°C in the following antibodies: β-tubulin III (β-tubIII, 1∶400, Covance), gamma amino-butyric acid (GABA, 1∶500, Sigma), glial fibrillary acidic protein (GFAP, mouse, 1∶500,Chemicon), GFAP (rabbit, 1∶500, Dako), glutamate (1∶500, Sigma), microtubular associated protein type 2 (MAP2, 1∶200, Sigma), Ki67 (1∶1.000, Novocastra), galactocerebroside C (GalC, 1∶300, Chemicon), gestin (1∶200, Chemicon), vimentin (1∶400, Immunological Sciences).

After rinsing in phosphate-buffered saline (PBS), cultures were incubated for 45 minutes at room temperature in the following secondary antibodies: Cy2 (against mouse or rabbit IgG, 1∶200, Jackson), Cy3 (against mouse or rabbit IgG, 1∶800, Jackson), Alexa 546 (against rabbit IgG or mouse IgG_1_, 1∶800, Molecular Probes), Alexa 488 (against rabbit IgG or mouse IgG_1_, 1∶800, Molecular Probes), Cy3 (against goat IgG, 1∶1000, Jackson). Nuclei were stained with DAPI. Data are reported as percentages of labeled cells over the total number of DAPI-labeled nuclei (at least 1500 nuclei per coverslip were scored)+/− the mean standard error (SE). Each value represents the average of three independent experiments unless specified.

Microphotographs were taken using a Zeiss Axiovert 200 direct epifluorescence microscope (Axioplan 2, Carl Zeiss, Jena, Germany), or with confocal microscope Leica TCS SP2 DMIRE2.

For mithocondria stain with JC1, IhNSC were plated onto laminin and after 4 hours cells were stained with JC1 (0.5 µM, Molecular Probes, Invitrogen) and Hoechst (50 mM, Sigma) without fixing with PFA. Microphotographs were taken using a Zeiss microscope.

Data are reported as percentages of labeled cells over the total number of nuclei±SEM. Each value represents the average of three independent experiments unless indicated into the legend.

### Western Blot Analysis

Immunoblots were performed as described [22] on total cell extracts prepared in Laemmli buffer (0.125 M Tris-HCl, pH 6.8, 5% SDS) containing 1mM phenylmethylsulfonyl fluoride (PMSF), 10 µg/mL pepstatin, 100 KIU/mL aprotinin, 10 µg/mL leupeptin (all from Calbiochem, San Diego, Calif.) and 1 mM Na_3_VO_4_. Extracts (50 µg protein/lane) plus 5% β-mercaptoethanol were electrophorezed using SDS-PAGE and electroblotted onto PVDF membranes (Millipore, Bedford, MA). Membranes were blocked with 4% non-fat dried milk or with BSA, incubated with monoclonal antibodies for p53 (Clone DO-7), nestin (Chemicon), β-actin and GAPDH (Sigma), and rabbit antibodies anti cleaved PARP, cleaved caspases 3 and cleaved caspases 9 (all from Cell Signaling, Boston, MA). PVDF membranes were incubated with antibodies in sealed bags using the X-blot roller hybridization instrument (Isenet, Milan, Italy). Binding of antibodies was detected with ECL Super Signal (Pierce, Rockford, IL) and bands quantified with ImageQuant.

### Cell Cycle Studies: BrdU/DNA Analysis and Detection

At 15–20 passages from the shift to the specific oxygen condition (1%, 2.5%, 5%, 20% O_2_), IhNSC were dissociated and kept in growing conditions for 24, 48 or 72 hours. Then cells were incubated with 20 µM 5-bromo-2-deoxyuridine (BrdU) for 20 min at 37°C, fixed in 70% ethanol and kept at 4°C before staining [23]. Fixed cells were washed with cold PBS and the DNA was denaturated with 1mL of 2N HCl for 20 min at RT. DNA denaturation was stopped by adding 3 mL 0.1 M sodium tetraborate pH 8.5. After centrifugation, the pellet was incubated with 1mL 0.5% (v/v) Tween-20 (Sigma, MO) in PBS containing 1% of BSA (Sigma) for 15 min at RT.

Cells were then incubated with anti-BrdU monoclonal antibody (BD Pharmingen, San Diego, CA) diluted 1∶10 in 0.5% (v/v) Tween-20 in PBS containing 1% of BSA for 60 min at RT in the dark. After centrifugation the pellet was incubated with 1mL of 0.5% (v/v) tween-20 in PBS 1% of BSA for 15 min at RT and then with Alexa 488 conjugated F(ab′)2 fragment goat anti-mouse IgG (Invitrogen, Carlsbad, CA), 1∶500 dilution in 0.5% (v/v) Tween-20 in PBS containing 1% of BSA) for 60 min at RT in the dark. The cells were then resuspended in 1 mL of a solution containing 2.5 µg/mL of propidium iodide (PI) in PBS and 7 µL RNAse 3 mg/mL in water, and stained overnight at 4°C in the dark. Biparametric BrdU/DNA analysis was done on at least 30000 cells for each sample by the FACSCalibur (BD Biosciences) and the data were analyzed using Summit 4.3 software.

### Changes in Mitochondrial Membrane Potential

Mitochondrial membrane potential was evaluated with the lipophilic cationic probe JC-1 (Invitrogen), which changes reversibly its color from green to orange as the membrane potential increases [24]. This property is due to the reversible formation of JC-1 aggregates upon membrane polarization that causes shifts in emitted light from 530 nm (i.e., emission of JC-1 monomeric form) to 590 nm (i.e., emission of J-aggregate) when excited at 490 nm. The apoptotic cells contain somewhat fewer JC-1 aggregates and more JC-1 monomers and their colour shifts from orange to green. Cells were stained with 0.5 µM JC-1 and kept at 37°C for 20 min, washed and resuspended in a total volume of 500 µL complete medium and analyzed by Cyan ADP (Coulter, Brea, CA).

### Terminal Deoxynucleotidyl Transferase-Mediated dUTP-FITC Nick-End Labeling Assay

Apoptosis was measured using the TUNEL assay kit (Roche Diagnostics, Basel, Switzerland) following the manufacturers' instruction for dual parameter flow cytometry. Analysis was performed on a FACSCalibur (BD Biosciences – CA USA) and the data were analyzed using Summit 4.3 software (Coulter).

### Statistical Analysis

Statistical analysis has been performed through ANOVA. Data are reported as means±SEM. Each value represents the average of three independent experiments, unless otherwise indicated in the legend. Data are considered statistically significant when p<0.01 unless otherwise indicated.

## Results

### Effects of Oxygen Concentrations on the Proliferation of IhNSC

**Figure 1:**
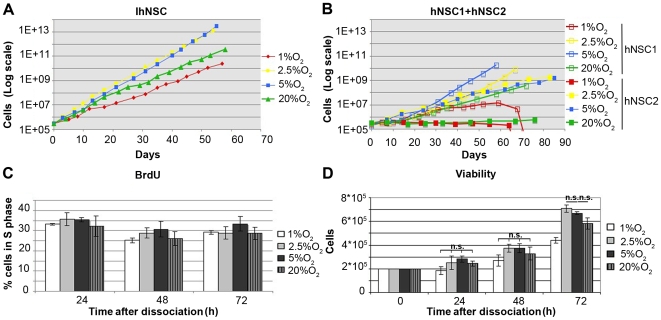
Mild hypoxia favors proliferation of IhNSC. (A, B) Graphs showing the proliferation rate of IhNSC (A) and hNSC lines 1 and 2 (B) when cultured at different concentrations of O_2_. At each passage only 2×10^5^ cells are plated while the logarithmic value of the total cell number is calculated on the base of amplification rate at each passage (see material and methods) and plotted against the days *in vitro* from the beginning of the experiment (N = 2; one representative curve is shown). As shown in this figure, in mild hypoxia, it could be possible to generate more than 1×10^13^ (IhNSC) or 1×10^10^ (hNSC) from 2×10^5^ cells after 60 days from the first passage. (C) Histogram showing the percentage of IhNSC in S phase (obtained through BrdU assay, see methods) at 24, 48, 72 hours after dissociation (N = 3) at each oxygen culture condition. Values are means±S.E.M. No significant differences were detected. (D) Histogram showing the number of viable IhNSC after dissociation at 0, 24, 48 and 72 hours after dissociation. For each oxygen condition 200.000 cells were plated (N = 2). Values are means±S.E.M. At 72 hours cells cultured in 2.5% and 5% O_2_ the percentage of viable IhNSCs was significantly higher than at 1% O_2_ (p<0.001). The difference among all the values at the different oxygen concentrations was statistically significant (P<0.01) unless indicated (*P<0.05, n.s. = not significant); one-way ANOVA followed by the Student's t-test.

IhNSCs were initially established and grown as neurospheres in 5% atmospheric oxygen (O_2_) [18]. For the present experiments, the cells were split after 45 passages to 1%, 2.5%, 5% and 20% O_2_ and continuously cultured in the respective oxygen conditions. In 2.5% and 5% of oxygen, IhNSC proliferated similarly and more rapidly than in 20% oxygen (Fig. 1A). Likewise, the proliferative rate of the non-immortalized parental hNSCs was maximal in mild hypoxia (2.5% and 5% oxygen) if compared to 20% oxygen, with minor variability among different cell lines (Fig. 1B). The hypoxic condition (1% O_2_) decreased the growth of IhNSCs and particularly of hNSCs, which eventually arrested and died. To determine whether the increased yield of precursors in mild hypoxia was due to augumented proliferation or reduced cell death or both, the DNA replication, detected by BrdU incorporation, and cell viability were simultaneously measured at different time points after dissociation. BrdU incorporation in cells during expansion did not appreciably vary at 24, 48 and 72 h from dissociation (Fig. 1C), and the fractions of cells in G1 and G2 phases were comparable among the different conditions (not shown), altogether indicating that cell cycle is not altered by O_2_. By contrast, the cell viability after dissociation was greater in mild hypoxia with respect to severe hypoxia (1% O_2_), becoming statistically significant after 72h from dissociation (Fig. 1D). Thus, the increased growth of IhNSCs under mild hypoxia appears to reflect a reduced apoptotic rate after dissociation, leading to the enhancement of self-renewal capacity.

### Survival of IhNSC at Different Concentrations of Oxygen

**Figure 2:**
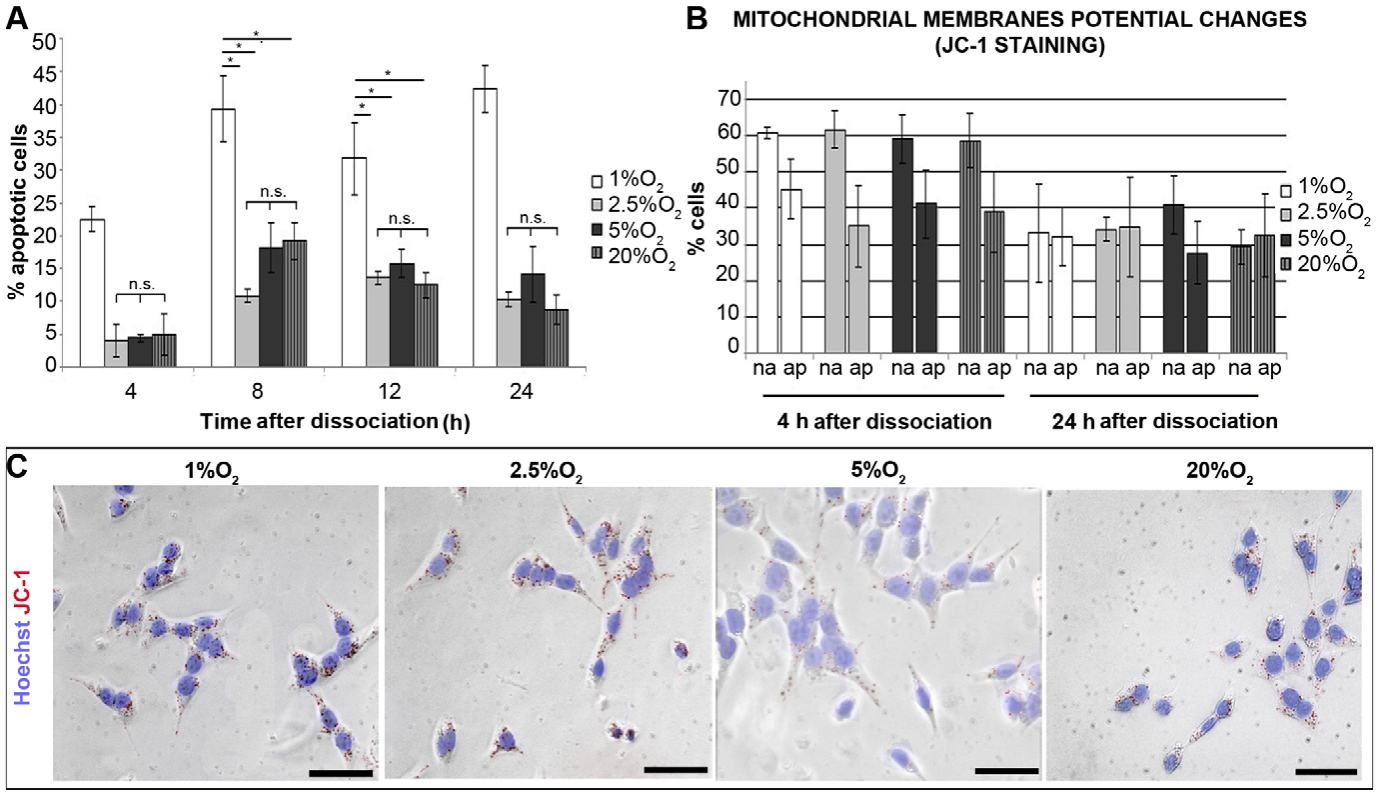
Survival of IhNSC at different O_2_ conditions. (A) Histogram showing the percentage of apoptotic cells (assessed by TUNEL assay, see methods) at 4, 8, 12 and 24 hours from dissociation (N = 3). Values are means±S.E.M. At 1% O_2_ the percentage of apoptotic cells in culture is significantly higher than at other conditions at each time point (p<0.001 at 4h). The difference among all the values at the different oxygen concentrations was statistically significant (P<0.01) unless indicated (*P<0.05, n.s., = not significant); one-way ANOVA followed by the Student's t-test. (B) Graph showing the percentages of non apoptotic (na) or apoptotic (ap) cells as assessed by JC1 staining assay for the detection of cells with depolarized mitochondrial potential, corresponding to apoptotic cells (N = 2). Values are means±S.E.M. No significant differences were detected between the different conditions. (C) Representative images of live dissociated IhNSC in 1%, 2.5%, 5% and 20% oxygen plated onto an adhesive substrate and stained with the mitochondrial and nuclear dyes JC1 (red) and Hoechst (blue) at 4 hours upon dissociation. The images were obtained by merging the bright field and fluorescent (for JC1 and Hoechst) pictures of the cells. Scale bars: 10µm.

To determine to what extent in mild hypoxic conditions the rate of survival after dissociation accounts for the increased growth rate over passages, we performed TUNEL analysis at 4, 8, 12 and 24h from dissociation. The highest percentage of apoptotic cells (39.33±5 at 8 h from dissociation) was found in 1% O_2_ with respect to the mild hypoxic (10.87±0.95 at 2.5% and 18.18±3.89 at 5% O_2_) and normoxic (19.2±2.85 at 20% O_2_) conditions (Fig. 2A), consistent with the previous data from the neurosphere and the viability assays (Fig. 1D).

**Figure 3:**
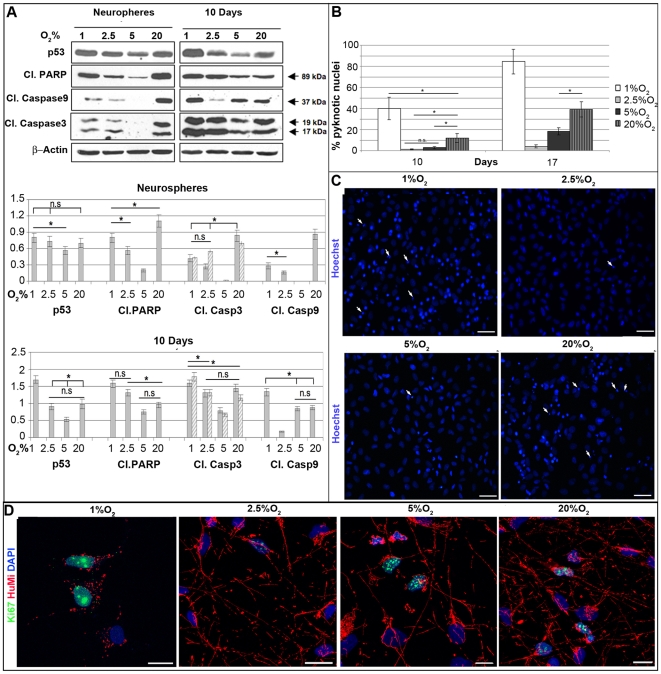
Survival and apoptosis during differentiation of IhNSC at different O_2_ conditions. (A) Western blot analysis of the expression of apoptotic markers at neurosphere stage and at 10 days differentiation. Lysates were prepared from undifferentiated and differentiated IhNSCs grown at the indicated oxygen concentrations and immunoblotted with antibodies specific for cleaved PARP, cleaved caspases 9 and 3, p53 and β-actin, the latter to normalize bands for equal loading of proteins per lane. Bands were quantified by densitometry analysis of the ECL-exposed films. (B) Pyknotic nuclei detected at 10 and 17 days of differentiation (N = 3). Values are means±S.E.M. The difference among all the values at the different oxygen concentrations was statistically significant (P<0.01) unless indicated with an asterisk (*P<0.05, n.s. = not significant); one-way ANOVA followed by the Student's t-test. (C) Representative images of pyknotic nuclei (white arrows, Hoechst stain, blue) at 17 days of differentiation. (D) Representative images of mithocondria (HuMi, red) at 17days of differentiation. Note proliferating cells (Ki67+, green) and the different distribution of mithocondria at 1% O_2_ relative to other O_2_ concentrations. Scale bars: 20µm (C), 10µm (D). For A and B, the differences at different oxygen concentrations was statistically significant (P<0.01) unless indicated (*P<0.05, n.s.: not significant); one-way ANOVA followed by the Student's t-test.

Because a decrease of oxygen concentration below the physiological threshold (1–5%) induces a metabolic shift from oxidative phosphorylation towards anaerobic glycolysis with the consequent diminution of mitochondria activity (revised in [25]), we assessed the mitochondrial activity of IhNSC cultured at different oxygen conditions and correlated it with the apoptotic score. IhNSCs were stained *in vivo* with JC-1 cationic dye indicator of mitochondrial membrane potential and analyzed by flow cytometry at 4 and 24 h from dissociation, but no significant differences were seen among the various oxygen conditions (Fig. 2B). These data, which were also confirmed by the fluorescence microscopy analysis of labeled samples (Fig. 2C, panel C), indicate that severe hypoxia (1% oxygen) compromises the survival but not the mitochondrial activity of IhNSCs. Since hypoxia induces a shift from mitochondrial respiration to anaerobic glycolysis, insufficient to fully support cell proliferation and differentiation, we evaluated the expression of apoptotic markers in IhNSC during differentiation by Western Blot analysis. As shown in Fig. 3A, the signals relative to cleaved PARP and cleaved caspases 3 and 9 were highest when neurospheres were grown at 1% or 20% O_2_. Similarly, cleaved PARP, cleaved Caspase 3 and 9 signals in IhNSCs undergoing differentiation were much greater in 1% O_2_ than in other oxygen concentrations (Fig. 3A). Interestingly, p53 accumulation correlated with increased levels of apoptosis. The frequency of pyknotic nuclei increased progressively during differentiation in all oxygen conditions (Fig. 3B, panel C), but the rate of cell death at 1% O_2_ (84.5±11.5%) was dramatically higher than at 2.5% (4±1.4), 5% (18.2±3.4) or 20% (39.1±7.2) O_2_ at 17 div (Fig. 3B). As previously shown for IhNSC during cell culture, mild hypoxia (2.5–5% O_2_) was associated with a modest level of apoptosis even during differentiation. Since an efficient differentiation is associated with an increase in aerobic metabolism and number of mitochondria [26], we determined the influence of low oxygen on mitochondrial patterning during differentiation. Differentiated IhNSC at 17 days were immunolabeled with anti-human mitochondria antibody and analyzed by confocal microscopy. Mitochondria aggregates were localized in the cell body and processes in IhNSC when cultured in mild hypoxia or normoxia (20% O_2_) with a “spaghetti-like” distribution (Fig. 3D), whereas severe hypoxic conditions caused a dramatic reduction of mitochondria per cell, with a perinuclear speckled pattern. Interestingly, an evident collapse of mitochondria morphology and location in the perinuclear area (Fig. 3D), a pattern typical of cells undergoing apoptosis [27].

### IhNSCs Generate Higher Percentages of Neurons in Mild Hypoxia

**Figure 4:**
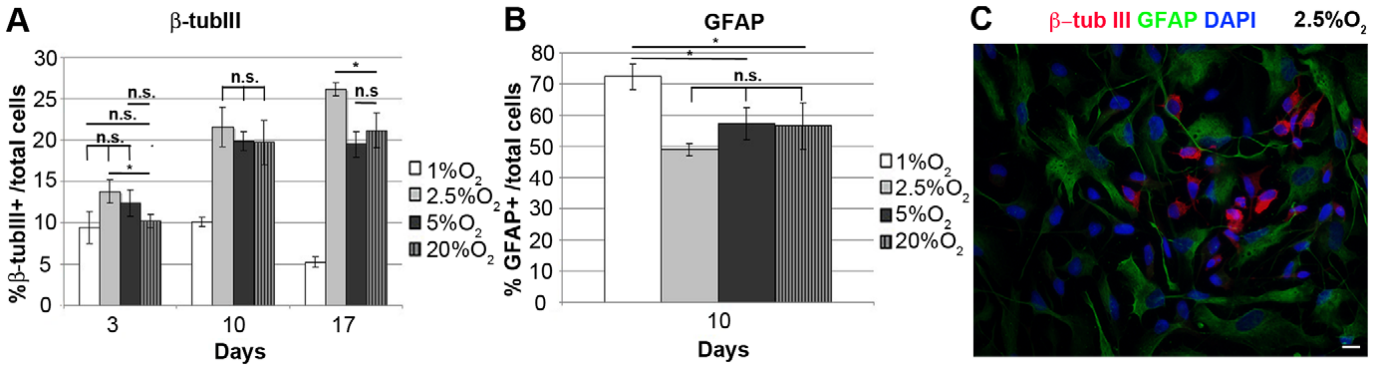
Mild hypoxia increases the percentage of IhNSC-derived neurons during in vitro differentiation. IhNSC were differentiated onto an adhesive substrate in medium without mitogens and fixed for immunocytochemical analysis after 3, 10 and 17 days *in vitro*. (A) Quantification of the percentage of neurons (β-tubIII+) over the total nuclei (DAPI+) number. Values are means±S.E.M (N = 3). Neurons were significantly less represented at 1% O_2_ with respect to the other O_2_ concentrations at 10 and 17 days *in vitro*. The difference among all the values at the different oxygen concentrations was statistically significant (P<0.01) unless indicated (*P<0.05), n.s., = not significant; one-way ANOVA followed by the Student's t-test.(B) Quantification of the percentage of astrocytes (GFAP+) over the total nuclei (DAPI+) number (N = 3). Values are means±S.E.M. At 10 days *in vitro* the percentage of astrocytes generated in 1% O_2_ was significantly (*p<0.05) higher with respect to the other conditions (1% O_2_ vs 2.5% O_2_, p<0.01). All other values were not significantly different (n.s., = not significant); one-way ANOVA followed by the Student's t-test. (C) Immunocytochemistry of differentiated cells showing the morphology of β-tubIII+ (red) and GFAP+ (green) cells in 2.5% oxygen at 10 days *in vitro*. Scale bar, 10µm.

Upon removal of mitogens, IhNSCs spontaneously undergo differentiation and concomitant proliferation arrest [18]. Considering the effects of oxygen levels on proliferating IhNSCs (described above), we analyzed the effects of oxygen on differentiation. The proportion of neural cell lineages derived from expanded precursors was determined by phenotypic analysis with antibodies against β-tubIII and MAP2 (specific for early and late differentiation stage of neuronal cells, respectively), GFAP (astrocytes), GalC (oligodendrocyte precursors). At 10 days of differentiation, the percentage of β-tubIII+ neurons derived from IhNSCs accounted for 19.7±2.7% at 20%, 19.9±1.1% at 5% and 21.6±2.4% at 2.5% O_2_, but for only 10.1±0.6% at 1% O_2_ (Fig. 4A). At 17 days, the β- tubIII+ neurons were higher at 2.5% O_2_ (26.1±0.8%) compared to 5% oxygen concentration (19.5±1.5%). On the other hand, the proportion of GFAP+ astrocytes was lower in 2.5% (49±1.9%), 5% (57.2±5.3%) and 20% (56.3±7.4%) compared with 1% O_2_ (72.3±4.1%) (Fig. 4B). The β-tubIII and GFAP never co-localized (Fig. 4C), indicating that the differentiation process of neuronal and astroglial cells was well-defined.

**Figure 5:**
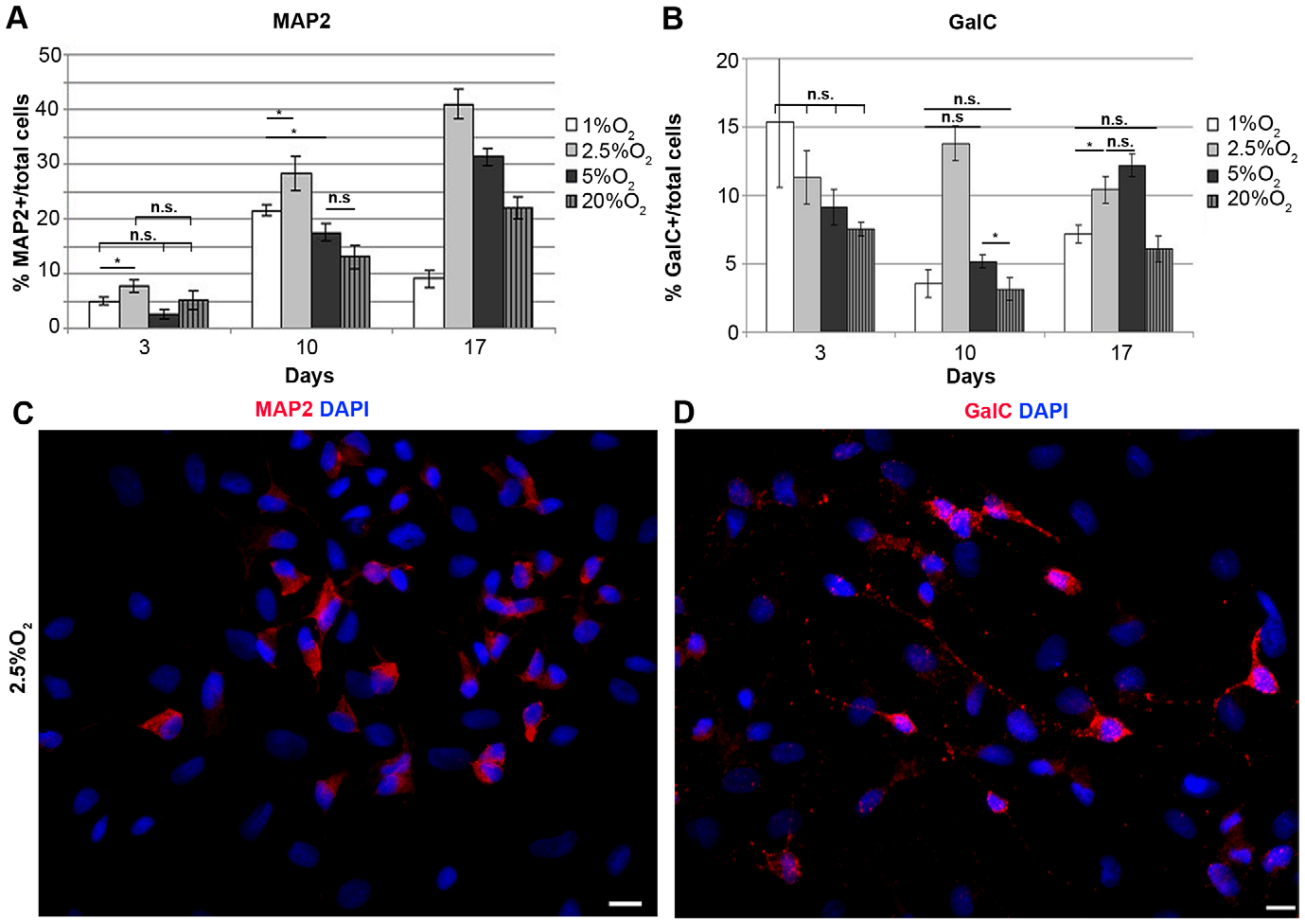
Mild hypoxia enhances the percentage of IhNSC-derived mature neurons and oligodendrocytes during *in vitro* differentiation. IhNSC were differentiated onto an adhesive substrate in medium without mitogens and fixed for immunocytochemical analysis at 3, 10 and 17 days. (A) Quantification of the percentage of mature neurons (MAP2+) over the total nuclei (DAPI+) number. Values are means±S.E.M (N = 3). At 10 and 17 days the percentages of MAP2+ neurons generated in 2.5% O_2_ were significantly higher with respect to the other conditions (B) Quantification of the percentage of oligodendrocytes (GalC+) over the total number of nuclei (DAPI+). Values are means±S.E.M (N = 3). At 10 days *in vitro* the percentages of GalC+ oligodendrocytess generated in 2.5% O_2_ were significantly higher with respect to the other conditions, while at 17days 2,5% and 5% O2 conditions generated comparable numbers of GalC+ cells. The differences among all the values at 1%, 2.5%, 5% and 20% oxygen was statistically significant (P<0.01) unless indicated (*P<0.05, n.s., = not significant); one-way ANOVA followed by the Student's t-test for all experiments. (C–D) Immunocytochemistry of differentiated cells showing the morphology of MAP2+ (red in C) and GalC+ (red in D) cells at 2.5% O_2_ at 10 days. Scale bars in C–D = 10µm.

To evaluate the expression of late neuronal proteins, differentiated IhNSCs were immunostained with the dendritic marker MAP2. At 10 days *in vitro*, 2.5% O_2_ induced the highest percentage of MAP2+ neurons (28.4±3.2%) relative to 20% (13±2.1%), 5% (17.6±1.5%) and 1% (21.5±1%) O_2_, correlating with the fractions of early β-tubIII+ neuronal cells. A similar tendency was observed at 17 days when the relative percentages of MAP2+ cells at 2.5%, 5% and 20% O_2_ were further increased, except for 1% O_2_ which determined a marked drop in MAP2+ cells (9±1.5%) (Fig. 5A and C).

IhNSCs generated GalC+ oligodendrocytes, with a greater number of these cells in mild hypoxia than in 1% or 20% O_2_. In particular, in 2.5% O_2_, they reached 13.8±1.3 already at 10 days of differentiation and appeared to survive at 17 days (11.5±1.3), while in 5% oligodendrocytes increased from 5.2±0.4 at 10 days to 14.3±1.8 at 17 days (Fig. 5B and D). The remaining cells were nestin positive (see below) or did not react with any of the markers tested.

### Mild Hypoxia Supports the Survival of Proliferating Neuronal and Oligodendroglial Progenitors

**Figure 6:**
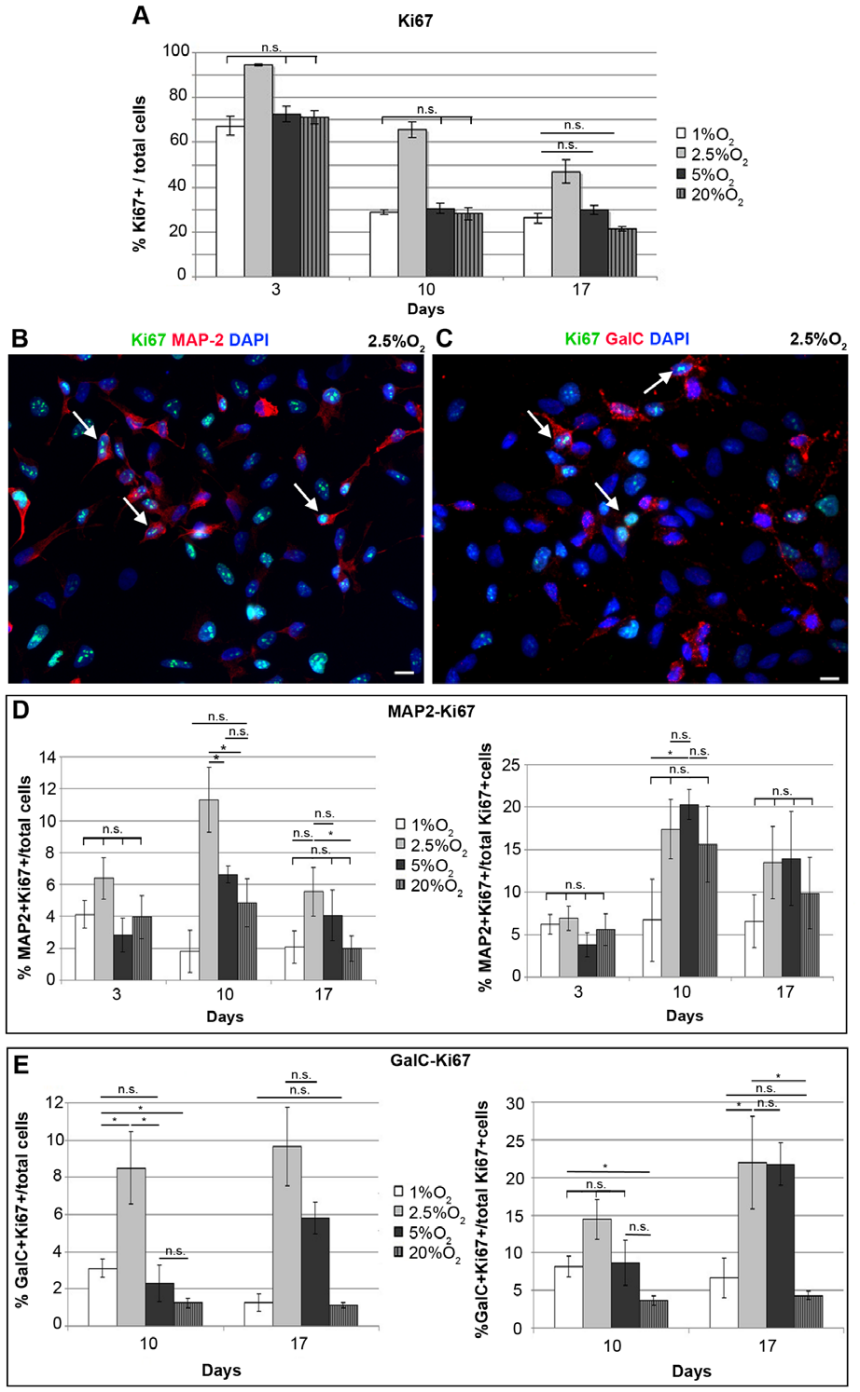
Mild hypoxia supports the survival of IhNSC-derived proliferating neuronal and oligodendroglial progenitors. IhNSCs were differentiated onto an adhesive substrate in medium without mitogens and fixed for immunocytochemical analysis at 3, 10 and 17 days. (A) Graph showing the percentage of proliferating (Ki67+) IhNSC cells over total nuclei (DAPI+) at 3, 10 and 17 days during *in vitro* differentiation. Values are means±S.E.M (N = 3). The 2.5% O_2_ resulted the most permissive condition to the proliferation of IhNSC-derived progenitors at each differentiation time. (B–C) Immunostaining showing the co-localization (arrows) between the nuclear proliferation marker Ki67 (green) with the neuronal MAP2 (red in B), and the oligodendroglial GalC (red in C) at 10 days. (D) Quantification of the percentage of proliferating neurons (MAP2+/Ki67+) over the total nuclei (DAPI+) number (left) and over the total of proliferating cells (Ki67+) number (right) at 3, 10 and 17 days. Values are means±S.E.M (N = 3). (E) Quantification of the percentage of proliferating oligodendrocytes (GalC+/Ki67+) over the total nuclei (DAPI+) number (left) and over the total of proliferating cells (Ki67+) number (right), at 10 and 17 days. Values are means±S.E.M (N = 3). The differences among all the values at 1%, 2.5%, 5% and 20% oxygen was statistically significant (P<0.01) unless indicated (*P<0.05, n.s., = not significant); one-way ANOVA followed by the Student's t-test for all experiments. Scale bars, (B–C) 10µm.

IhNSCs undergo a gradual proliferative arrest during differentiation [18]. To assess to what extent residual proliferation of differentiating IhNSCs is influenced by oxygen levels, we estimated the fraction of IhNSC expressing the S-phase marker Ki67. Consistent with the counts of apoptotic nuclei during differentiation (Fig. 3B), albeit the percentage of Ki67+ cells decreased with time, IhNSCs grown in 2.5% O_2_ contained the largest fraction of Ki67+ cells (65.6±3.5% at 10 days), consistently higher than at other oxygen concentrations (Fig. 6A).

We further investigated if a selective prolonged survival of neuronal and oligodendroglial progenitors could account for the greater percentage of β-tubIII+ (or MAP2+) and GalC+ cells, generated during differentiation at mild hypoxic conditions (Figs. 4A, 5A and B). To this aim, differentiating cells we co-labeled with Ki67 and MAP2 (Fig. 6B) or GalC (Fig. 6C). As expected, in 2.5% oxygen IhNSC generated a higher percentage of proliferating neuronal progenitors compared to 1%, 5% and 20% O_2_. Indeed, at 10 days 11.3±2% of the cells in 2.5% oxygen was co-expressing Ki67 and MAP2, relative to 6.6±0.4 in 5%, 1.8±1.3 in 1% and 4.9±1.5 in 20% O_2_ (Fig. 6D, left).

A similar trend was evident for the absolute number of proliferating oligodendroglial progenitors: at 10 days, 8.5±2% of the cells in 2.5% oxygen co-expressed Ki67+ and GalC, compared to 2.7±1 in 5%, 3.1±0.5 in 1% and 1.2±0.3 in 20% O_2_ (Fig. 6E, left). Interestingly, of the total number of Ki67+ cells at 17 days in 2.5 and 5% O_2_, the neuronal MAP2+/Ki67+ (Fig. 6D, right) and oligodendroglial GalC+/Ki67+ (Fig. 6E, right) accounted for 14% and 22%, respectively, indicating that mild hypoxia is optimal for survival and differentiation.

### Oxygen Regulates Neurotransmitter Phenotypes of IhNSC-Derived Neurons

**Figure 7:**
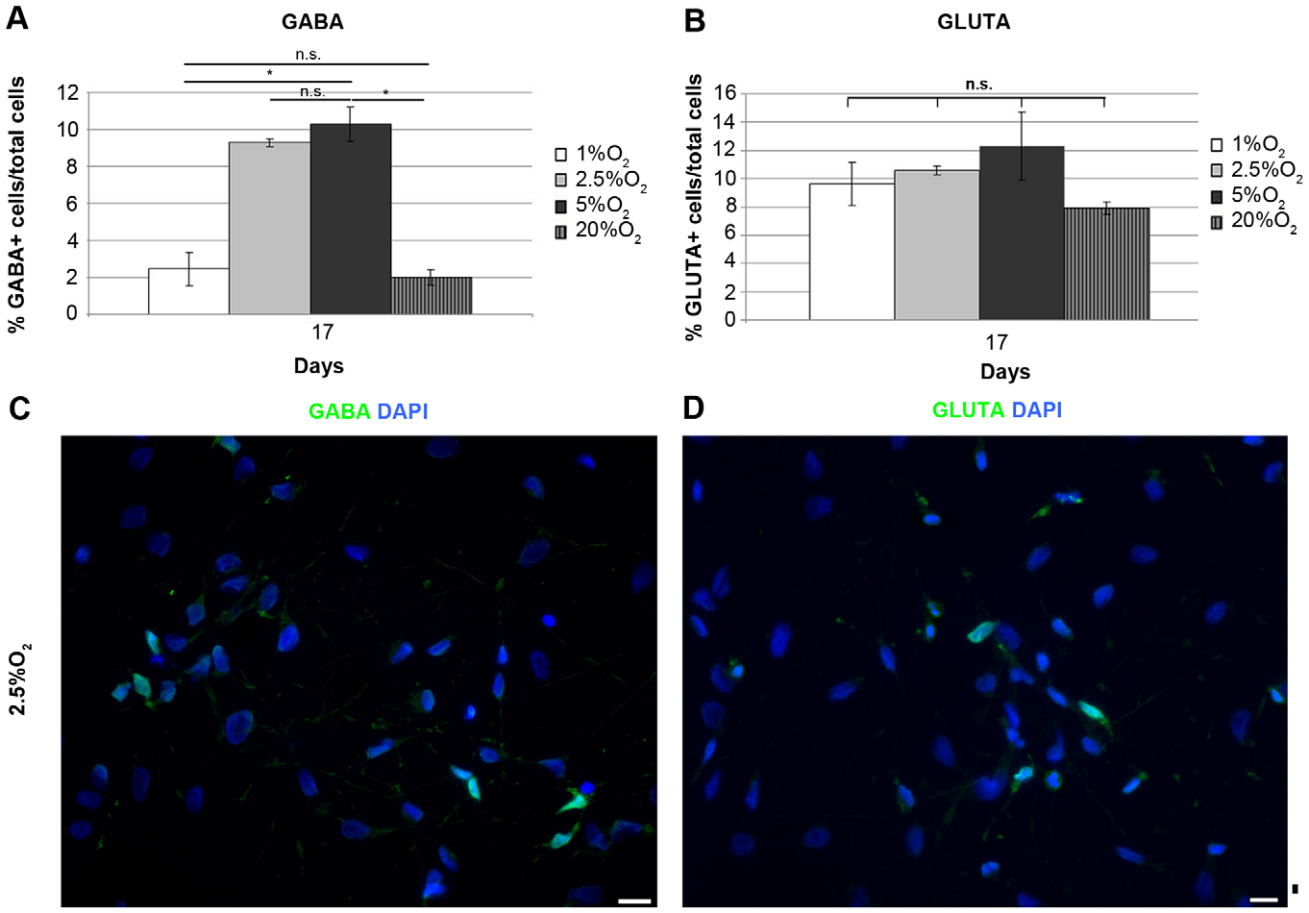
Oxygen regulates neurotrasmitter phenotypes of IhNSC-derived neurons. IhNSCs were differentiated onto an adhesive substrate in medium without mitogens and fixed for immunocytochemical analysis at 3, 10 and 17 days. (A) Quantification of the percentage of GABAergic neurons (GABA+) over the total nuclei (DAPI+) number, at 17 days. Values are means±S.E.M (N = 3). At 17 days, the percentage of GABA+ cells significantly decreased in 1% O_2_ and 20% O_2_ with respect to mild hypoxia conditions. (B) Quantification of the percentage of glutamatergic neurons (GLUTA+) over the total nuclei (DAPI+) number at 17 days. Values are means±S.E.M (N = 3). At 17 days the percentage of GLUTA+ cells was significantly higher in hypoxic conditions with respect to 20% O_2_. The differences among all the values at 1%, 2.5%, 5% and 20% oxygen was statistically significant (P<0.01) unless indicated (*P<0.05, n.s., = not significant); one-way ANOVA followed by the Student's t-test for all experiments. (C–D) Immunostaining showing the morphology of GABA+ cells (C, green) and GLUTA+ cells (D, green) in 2.5% O_2_ at 17 days. Scale bars 10µm.

Recently, we have shown that in 5% O_2_ IhNSCs generate GABAergic and glutamatergic neurons, and only few tyrosine hydroxylase (TH)+ neurons [18]. Since mild hypoxia enhances IhNSC neurogenesis, we examined the effect of oxygen on neuronal differentiation into GABA, glutamatergic, catecholaminergic (TH), cholinergic (ChAT) and serotoninergic (5′-HT) subtypes. At 17 days, the GABA+ cells accounted for 10.3±0.9 and 9.3±0.2% in 5% and 2.5% O_2_ respectively, but significantly decreased to 2.4±0.9 in 1% O_2_ and 2±0.4 in 20% O_2_ (Fig. 7A and C). On the other hand, we observed a tendency of the fraction of glutamatergic neurons to be maximal in severe and mild hypoxic conditions (9.6±1.5% in 1% O_2_; 10.6±0.3% in 2.5% O_2_ and 12.3±2.4% in 5% O_2_) and slightly decreased with increasing oxygen concentrations (7.9±0.4% in 20% O_2_) (Fig. 7B and D), consistent with previous studies [14]. As hypoxia increases mesencephalic differentiation into TH+ neurons [16], [17] we tested if also telencephalic-diencephalic precursors could acquire a dopaminergic phenotype by lowered oxygen. Of note, only 1% of differentiated IhNSCs are TH+ when cultured in 5% oxygen [18]. We found that no TH+ cells were generated in 20% O_2_, and percentages comparable to 5% oxygen were obtained under 2.5% and 1% O_2_. Sporadic cholinergic (ChAT+) neurons were observed in mild hypoxia, whereas 5′-HT+ neuronal cells were never detected.

### Severe Hypoxia Impairs Differentiation of IhNSCs

**Figure 8:**
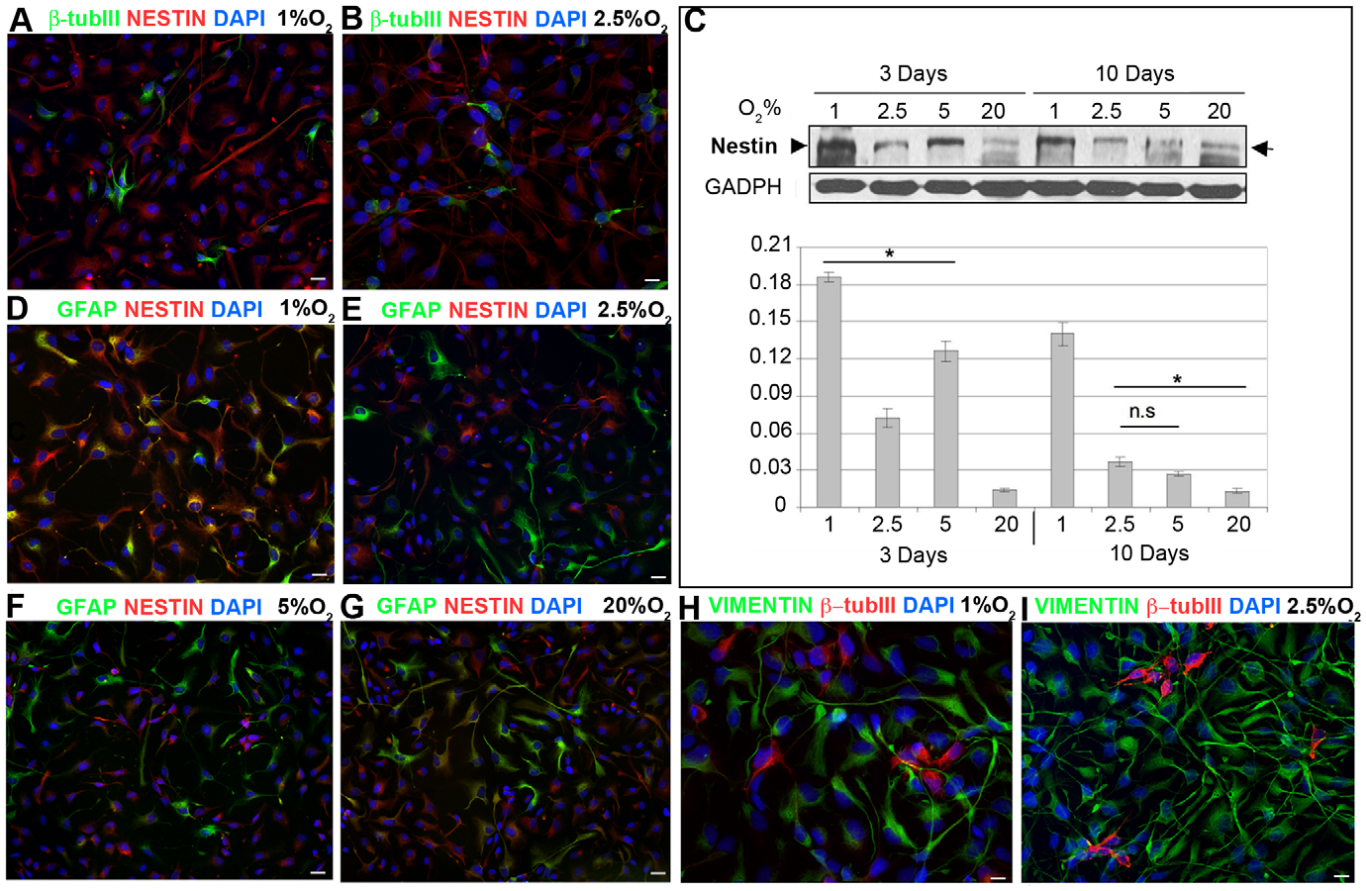
Hypoxia impairs differentiation of IhNSCs. IhNSCs were differentiated onto an adhesive substrate in medium without mitogens and fixed for immunocytochemical analysis at 3, 10 and 17 days. (A–B) Immunostaining showing the non co-localization between the neuronal marker β-tubIII (green) and nestin (red) in IhNSC cells at 10 days in 1% (A) and 2,5% (B) O_2_. (C) Western blot analysis of the variation of nestin (arrows), during differentiation. Lysates were prepared from IhNSCs grown and differentiated for 3 and 10 days at the indicated oxygen concentrations. Lysates were immunoblotted with antibodies specific for nestin and GADPH, the latter to normalize bands for equal loading of proteins per lane. Bands were quantified by densitometry analysis of the ECL-exposed films. (D–G) Immunostaining showing the co-localization (D, yellow) between GFAP (green) and nestin in cells at 10 days in 1% O_2_. The two markers never showed colocalization at 2,5% (E) and 5% (F) O_2_ at the same time point, and only few cells were co-expressing the two markers at 20% O_2_ (G, yellow). (H–I) Immunostaining showing the non co-localization between the neuronal marker β-tubIII (red) and vimentin (green) in IhNSC cells at 10 days in 1% (H) and 2,5% (I) O_2_. Scale bars, (A–B, D–G) 20µm, (H,I) 10 µm.

Hypoxia promotes an undifferentiated state in several primitive and precursor cell populations [28]. As lower percentages of neuronal and glial cells were generated from IhNSCs cultured at 1% O_2_, we searched for the presence of quiescent or immature progenitors being unable to reach terminal differentiation. To this aim, the intermediate filament nestin (Fig. 8 A–G) and vimentin (Fig. 8 H–I) were used to discriminate stem and progenitor cells from the differentiated progeny, as reported [29]. Nestin expression progressively decreased with differentiation in mild hypoxia, to the least extent in 1% O_2_, as revealed by the greater number of nestin+ cells detected by immunofluorescence (Fig. S1) and Western blot (Fig. 8C). No co-expression of nestin and β-tubIII was detected (Fig. 8A and B), indicating that nestin+ cells in 1% O_2_ do not become immature neuroblasts. Bona fide NSCs *in vivo* [7] are positive for both nestin and GFAP. These markers were co-expressed in many cells in 1% O_2_ (Fig. 8D), and only in a small fraction in 20% O_2_ (Fig. 8G), suggesting an incomplete differentiation process in stringent oxygen culture conditions. To exclude that this effect could reflect an aberrant commitment of IhNSC-derived progenitors to the astrocytic lineage, we assessed the expression of vimentin, an early astroglial marker, together with the early neuronal marker β-tubIII. While all cultures contained a consistent number of vimentin+ cells, none co-expressed β-tubIII (Fig. 8H and I). These experiments thus indicate that hypoxia influences the differentiation capacity of IhNSC, forcing the progenitors into a quiescent state.

## Discussion

Oxygen plays a central role in regulating CNS progenitor cell proliferation, development and homeostasis. In the mammalian brain, interstitial tissue O_2_ levels range from about 0.55 to 8% [15] and there is evidence that control of O_2_ availability is central to the normal architecture of CNS and underlies the etiology of several neurological diseases. Here we have shown that mild hypoxia (2.5–5% O_2_), which better approximates the physiological setting, promotes survival of actively replicating IhNSCs as well as the yield of oligodendroglial and neuronal cells.

### Proliferation and Survival in Hypoxic Conditions

Several studies show that low oxygen concentrations (1–5%) allow cell proliferation through adapted mitochondrial respiration [30], [31]. Under anoxic conditions, mitochondrial respiration is inhibited and the energy is provided by anaerobic glycolysis, energetically insufficient to fully support cell proliferation and differentiation [25]. Here, we have demonstrated that O_2_ concentration is critical for the growth and survival of IhNSCs. Mild hypoxia in particular, enhanced the proliferation of IhNSCs, compared with 1% and 20% O_2_, in agreement with previous data on neural crest stem cells, neuronal progenitors or mesencephalic precursor cells [12], [16], [17], [32]. In accordance to these findings, Yoshida et al [33] have recently shown that mild hypoxia is the optimal condition for both the generation and proliferation of induced pluripotent stem cells (iPS) from human somatic cells in comparison with 1% or 21% O_2_, leading respectively to cytotoxic effects or to a less efficient reprogramming process. Indeed, in our study the neurosphere assay showed that in marked contrast to 2.5–5% O_2_, 1% O_2_ decreased the proliferation of IhNSCs and raised the rate apoptotic, when compared to 20% O_2_. In comparison with immortalized IhNSC [18], parental hNSCs were even unable to proliferate in 1% O_2_, dying after few passages, presumably because of cell cycle arrest and inhibition of transcriptional activity [34]–[36]. Thus, IhNSC provided a unique tool to study the effects of severe hypoxia on hNSC self-renewal and multipotency.

The proliferation rate of NSCs as determined by the neurosphere assay represents the output of two variables i) the cell cycle kinetics of the surviving precursors, ii) the rate of survival after dissociation. To elucidate at what extent oxygen could affect the cell cycle, we performed BrdU incorporation assay, but no major differences in the fraction of S-phase cells were detected among IhNSCs cultured at various O_2_ concentrations. Furthermore, viability assay showed a significant fraction of dead cells at 1% and 20% O_2_, suggesting an enhancement of self-renewal capacity in mild hypoxia. Taking together, the BrdU incorporation and cell viability data, and applying Steel's formula, we found no appreciable differences in the average cycling times of IhNSCs cultured at the diverse oxygen conditions, leading to conclude that oxygen concentration affects IhNSC survival but not the cell cycle kinetics. To gain further insight into IhNSC survival after dissociation, we quantified apoptotic cells by TUNEL analysis and found a dramatic increase of cell death in 1% oxygen, which evidently accounts for the slow growth curve of neurospheres. In view of this, we postulated that a less efficient mitochondrial activity at 1% oxygen could contribute to severe hypoxia-induced apoptosis [37]. To test this hypothesis, we investigated the mitochondria activity by JC1 staining assay and unexpectedly found no impairment at 1% with respect to 2.5, 5 and 20% oxygen. Furthermore, the amount of mitochondrial JC1 aggregates as detected by fluorescence microscopy in 1% was comparable to 2.5%, 5% and 20% oxygen.

### Cell Survival during Differentiation

We demonstrated that oxygen concentration is also critical for the neural differentiation of IhNSCs. In marked contrast to 2.5 and 5% O_2_, 1% O_2_ decreased the neural differentiation potential and increased the cell death also when compared with 20% O_2_.We evaluated the rate of survival of IhNSCs during differentiation by counting the pyknotic nuclei and found a significantly higher percentage of apoptotic cells at 1% oxygen, consistent with previous studies [14] showing that severe hypoxia corresponds to pathopysiological conditions as in cerebral ischemia [38]. Considering that differentiation is associated with increased amount of mitochondria and increased aerobic metabolism [39], [40], we investigated the mitochondria patterning during differentiation. At 1% oxygen the mitochondrial aggregates showed a perinuclear localization and pseudo-globular and speckled morphology at 17 days during differentiation, typical of mitochondrial fission events correlated with cell death or degeneration [27]. In marked contrast under mild hypoxia and normoxia the mitochondria appeared fused and showed ‘Spaghetti-like’ pattern throughout the cell body and processes.

We further investigated the multipotency of IhNSC, and as expected, the neuronal differentiation was enhanced under mild hypoxia, especially at 2.5% O_2_ which yielded significantly higher proportions of both β-tubIII+ and MAP2+ cells when compared with 1% and 20% O_2_. Thus it seems to be a common phenomenon that proliferation and differentiation of NSCs or neuronal progenitor cells is higher under physiological conditions than under non-physiologically high or low oxygen.

We have shown, in accordance with previous studies [16], [41], [42], that culturing cells in physiologically normal (2.5–5% O_2_) than non-physiologically high (20% O_2_) or low (1% O_2_) conditions drastically enhance neuronal and oligodendroglial differentiation of hNSC. In particular, at 2.5% O_2_ IhNSC generated the highest percentages of neuronal cells (up to 28% of β-tubIII+ and 44% of MAP2+). Consistent with previous studies [14], we observed an increase of GABAergic and to less extent of glutamatergic neuronal phenotypes under mild hypoxia.

Oxygen concentration has been implicated in controlling oligodendrocyte progenitor proliferation and survival [43]. O2A progenitors with a more reduced state have a greater likelihood of self-renewal compared to cells with a more oxidized state. We addressed the role of oxygen in oligodendroglial differentiation and showed a marked increase of GalC+ oligodendrocytes in mild hypoxia (up to 15.5%) compared to other O_2_ concentrations. The analysis of apoptosis during differentiation suggests that an enhancement of the survival of IhNSC-derived progenitors could account for the increase of the neurogenic/oligodendrogenic potential. It is well established that neuronal and oligodendroglial cells are characterized by an higher threshold of vulnerability to hypoxic-ischemic injury with respect to the astroglial phenotype [9]. Indeed, we observed an increase of GFAP+ cells generated by differentiation of IhNSC in 1% O_2_, with a concomitant decrease of neuronal and oligodendroglial cells relative to higher oxygen concentrations. Moreover, the analysis of early markers for immature progenitors showed that at 1% O_2_ nestin expression was only slightly down-regulated with differentiation and that a significant fraction of cells co-expressed nestin and GFAP whereas faint levels of nestin were detectable at other oxygen concentrations. This observation supports the proposal that self-renewing divisions of stem cells are conditioned by hypoxic metabolic type [25].

In our study, a major apoptotic tendency of IhNSCs undergoing differentiation at 1% oxygen is probably paralleled by the rest of surviving NSC in a state of quiescence [44] where they remain GFAP+/nestin+ and with low mitochondrial content, the latter is typical of severe hypoxic metabolic condition [39], [40]. We have also excluded that any aberrant event could account for the presence of nestin+/GFAP+ cells during differentiation at 1% oxygen, by checking the expression of early markers specific for neuronal and astroglial lineages. The non co-localization of nestin with β-tubIII and of vimentin with β-tubIII supports the identification of a major fraction of nestin+/GFAP+ IhNSCs with quiescent progenitors.

In conclusion, our study provides evidence that mild hypoxia, known to occur in the NSC niches of the adult brain, increases proliferation, reduces cell death and enhances neuronal and oligodendroglial differentiation of hNSC. These findings represent an important advance for studies on brain injuries like stroke and for the *ex vivo* generation of specific neurons and oligodendrocytes for neurodegenerative diseases.

## Supporting Information

Figure S1 Severe hypoxia impairs differentiation of immortalized human neural stem cells (IhNSCs). IhNSCs were differentiated onto an adhesive substrate in medium without mitogens and fixed for immunocytochemical analysis at ten days. The graph shows the percentage of early undifferentiated IhNSCs (nestin+) over total nuclei (DAPI+ cells) at ten days during in vitro differentiation. Values are means±S.E.M (N = 3). In 1% O_2_, the majority of the cells are immature nestin+ progenitors. The differences among all the values at 1%, 2.5%, 5%, and 20% oxygen was statistically significant (P<0.01) unless indicated (*P<0.05, n.s. = not significant); one-way ANOVA followed by the Student's t-test.
(1.84 MB TIF)
