# Supplementary figures and images for: Mild Hypoxia Enhances Proliferation and Multipotency of Human Neural Stem Cells
Source: PLoS One. 2010 Jan 5;5(1):e8575. doi: 10.1371/journal.pone.0008575 (PMC2797394; doi:10.1371/journal.pone.0008575)

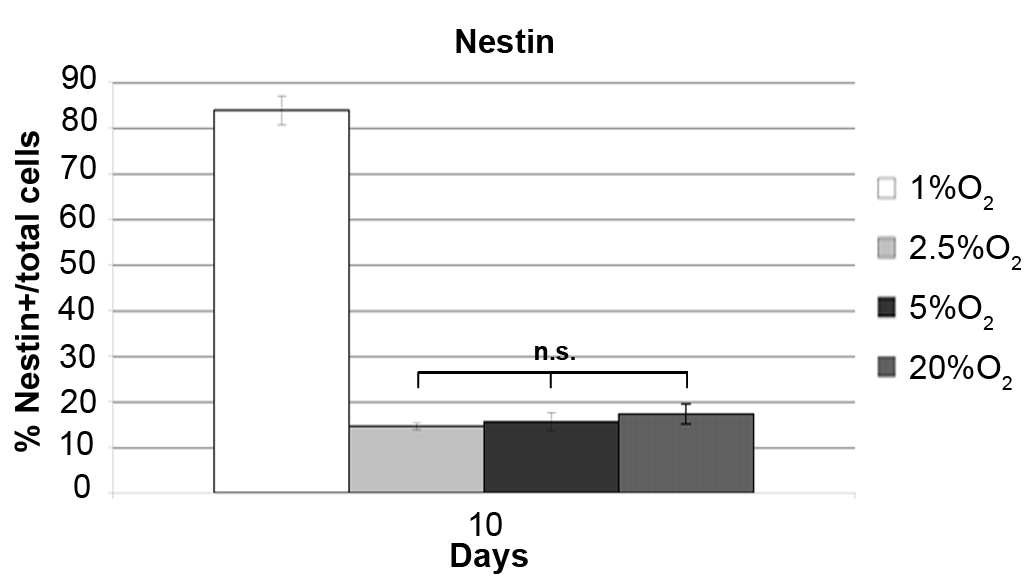

Supplement: Figure S1 — Severe hypoxia impairs differentiation of immortalized human neural stem cells (IhNSCs). IhNSCs were differentiated onto an adhesive substrate in medium without mitogens and fixed for immunocytochemical analysis at ten days. The graph shows the percentage of early undifferentiated IhNSCs (nestin+) over total nuclei (DAPI+ cells) at ten days during in vitro differentiation. Values are means±S.E.M (N = 3). In 1% O2, the majority of the cells are immature nestin+ progenitors. The differences among all the values at 1%, 2.5%, 5%, and 20% oxygen was statistically significant (P<0.01) unless indicated (*P<0.05, n.s. = not significant); one-way ANOVA followed by the Student's t-test. (1.84 MB TIF) [file pone.0008575.s001.tif]
